# Therapeutic effects of anti-HMGB1 monoclonal antibody on pilocarpine-induced status epilepticus in mice

**DOI:** 10.1038/s41598-017-01325-y

**Published:** 2017-04-26

**Authors:** Li Fu, Keyue Liu, Hidenori Wake, Kiyoshi Teshigawara, Tadashi Yoshino, Hideo Takahashi, Shuji Mori, Masahiro Nishibori

**Affiliations:** 1 Department of Pharmacology, Okayama University Graduate School of Medicine, Dentistry, and Pharmaceutical Sciences, Okayama, 700-8558 Japan; 2 Department of Pathology, Okayama University Graduate School of Medicine, Dentistry, and Pharmaceutical Sciences, Okayama, 700-8558 Japan; 3 Department of Pharmacology, Kinki University School of Medicine, Osakasayama, 589-8511 Japan; 4 School of Pharmacy, Pharmacology, Shujitsu University, Okayama, 703-8516 Japan

## Abstract

Inflammatory processes in brain tissue have been described in human epilepsy of various etiologies and in experimental models of seizures. High mobility group box-1 (HMGB1) is now recognized as representative of damage-associated molecular patterns (DAMPs). In the present study, we focused on whether anti-HMGB1 antibody treatment could relieve status epilepticus- triggered BBB breakdown and inflammation response in addition to the seizure behavior itself. Pilocarpine and methyl-scopolamine were used to establish the acute seizure model. Anti-HMGB1 mAb showed inhibitory effects on leakage of the BBB, and on the HMGB1 translocation induced by pilocarpine. The expression of inflammation-related factors, such as MCP-1, CXCL-1, TLR-4, and IL-6 in hippocampus and cerebral cortex were down-regulated by anti-HMGB1 mAb associated with the number of activated astrocytes, microglial cells as well as the expression of IL-1β. Both hematoxylin & eosin and TUNEL staining showed that the apoptotic cells could be reduced after anti-HMGB1 mAb treatment. The onset and latency of Racine stage five were significantly prolonged in the anti-HMGB1 mAb group. These results suggested that anti-HMGB1 mAb prevented the BBB permeability, reduced HMGB1 translocation while inhibiting the expression of inflammation-related factors, protected against neural cell apoptosis and prolonged Racine stage 5 seizure onset and latency.

## Introduction

Epilepsy is a disabling neurological disorder affects around 60 million people of all ages worldwide^1, 2^. In about 30% of affected individuals, epilepsy is refractory to pharmacological treatment and if not treated well could lead to brain damage or death^3^. The mechanism involved in the pathogenesis of epilepsy is not well understood^4–6^. Temporal lobe epilepsy (TLE) is the most common form of partial epilepsy, affecting at least 20% of all seizure patients^7^. Pilocarpine-induced epilepsy is a well-established animal model for status epilepticus (SE) and has also been reported as a good candidate for human TLE^8^.

Some models of epilepsy have revealed neuronal cell damage and loss in the CA1 and CA3 regions and dentate gyrus of the hippocampus^9^. A recent study on TLE also indicated atrophy, neuronal loss and gliosis in the DG, CA1, and CA3 regions, the entorhinal cortex and the amygdala^10^. Epilepsy is often associated with breakdown of the blood-brain barrier (BBB) altered peripheral immune response, and neuronal network reorganization^11–15^. Inflammatory processes in brain tissue have been described in human epilepsy of various etiologies and in experimental models of seizures. Indeed, recent data suggest that inflammation may play an important role in the pathogenesis of epilepsy^6^.

HMGB1, a non-histone DNA-binding protein, has been suggested to play important roles in the regulation of gene expression, DNA repair, and maintenance of chromatin structure. HMGB1 shares 100% amino acid sequence identity between mice and rats, and a 99% homology between rodents and humans^16, 17^. Once the tissues are damaged by any of several causes, HMGB1 might be released from necrotic cells passively, as well as from living cells, including macrophages, neurons^18^, astrocytes^19^ and hepatocytes^20^. In ischemic^21^ or traumatic brain injury^22^, the HMGB1 released from the neuronal nucleus induces a contractile response in pericytes and vascular endothelial cells and disrupts the integrity of the BBB, leading to the augmentation of brain inflammatory responses.

Thus, HMGB1 appears to be a mediator of the neurovascular unit, and excessive HMGB1 release may be associated with brain injury and dysfunction^23^. *Maroso et al*.^24^ showed that kainate injection into the hippocampus induced ictogenesis through the release of HMGB1 and TLR-4 stimulation, at the same time increased expression of HMGB1 had been found in human and rat epileptic brain. Therefore, we hypothesized that HMGB1, as a pro-inflammatory cytokine-like molecule, might be involved in the development of epileptogenesis, especially through BBB disruption and induction of inflammatory processes. This study was designed to determine whether HMGB1 is involved in the pathogenesis of epilepsy or the ability of anti-HMGB1 mAb to protect BBB permeability, neurons and inflammation.

## Results

### Effects of anti-HMGB1 mAb on BBB integrity under an acute epileptic state

**Figure 1:**
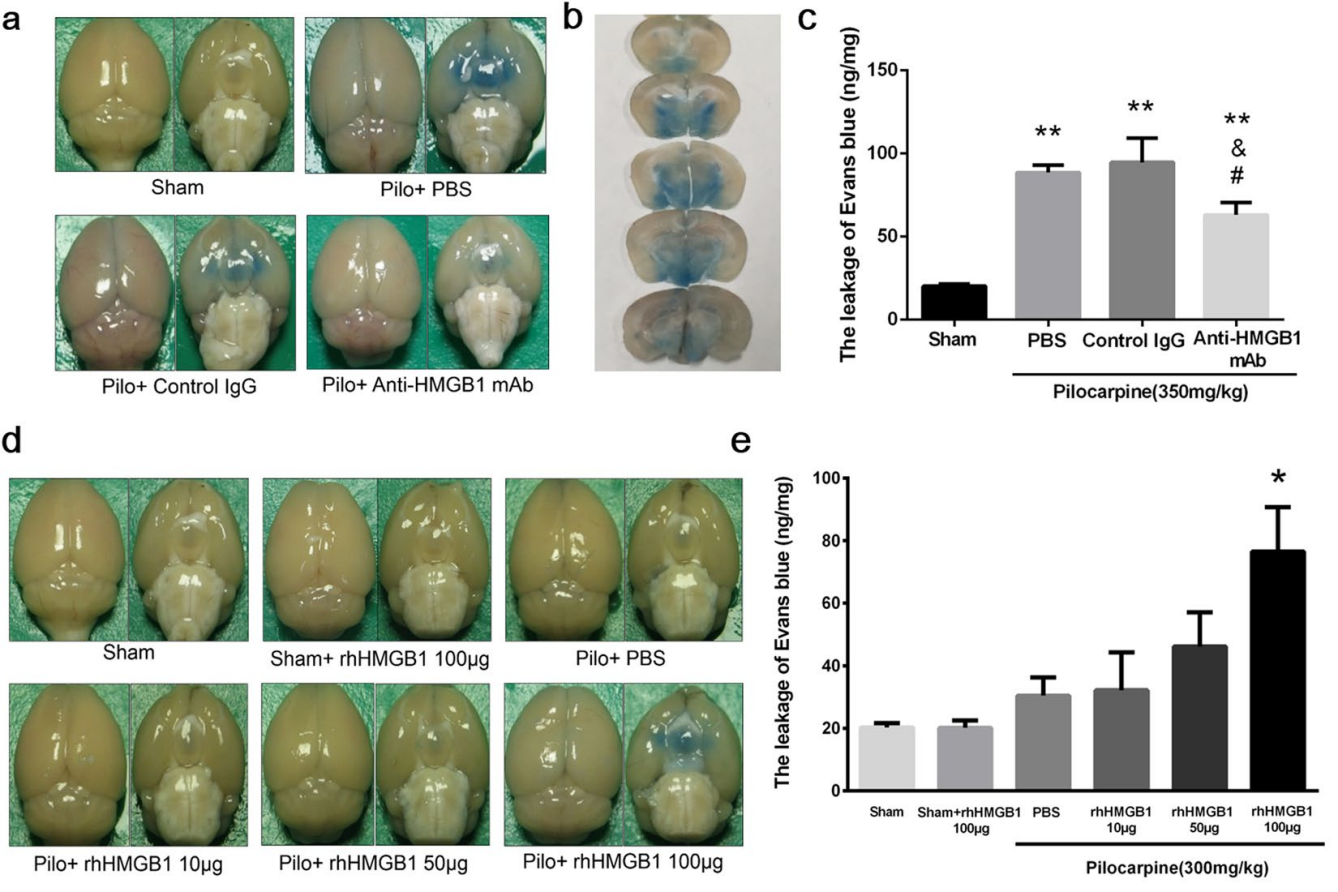
Effect of anti-HMGB1 mAb on BBB permeability in pilocarpine-induced acute status epilepticus. The permeability of brain capillary vessels was examined by intravenously injecting Evans blue (100 mg/kg, i.v.). Anti-HMGB1 mAb (1 mg/kg), control IgG, PBS was administered intravenously to mice which reached Racine stage 5 after onset of epilepsy (**a**). The distribution of Evans blue in brain were significantly shown in thalamus and hypothalamus region (**b**). The leakage of Evans blue into the brain parenchyma was quantified at 4 h after anti-HMGB1, control IgG or PBS injection, and the results are expressed as the means ± SEM of 5 mice. **p < 0.01, ^&^p < 0.05 and ^#^p < 0.05 vs. Sham, PBS and Control IgG, respectively (**c**). The effects of intravenous administration of different concentrations of exogenous rhHMGB1 on BBB permeability after pilocarpine treatment are shown (**d**). The results were expressed as the means ± SEM of 7 mice. *p < 0.05 compared with Pilo + HMGB1 100 μg (**e**).

As shown in Fig. 1a, pilocarpine-induced acute status epilepticus dramatically increased the leakage of Evans blue, especially into the thalamus and hypothalamus regions (Fig. 1b). However, after anti-HMGB1 mAb treatment the concentration of Evans blue dye decreased during pilocarpine-induced acute status epilepticus, while the control IgG group did not show any reduction in BBB leakage. The quantitative analysis revealed that the Evans blue levels were significantly increased in the PBS and control IgG groups compared to the sham and anti-HMGB1 treatment groups.

Injection of recombinant human HMGB1 after pilocarpine (300 mg/kg) enhanced the leakage of Evans blue dye in an HMGB1 dose-dependent manner (Fig. 1d). This result indicated that HMGB1 would promoted the BBB breakdown under an epileptic state. Quantification analysis indicated that the i.v. administration of exogenous recombinant human HMGB1 to mice enhanced the pilocarpine-induced BBB permeability in a dose-dependent manner.

### Detection of HMGB1 dynamics after pilocarpine injection

**Figure 2:**
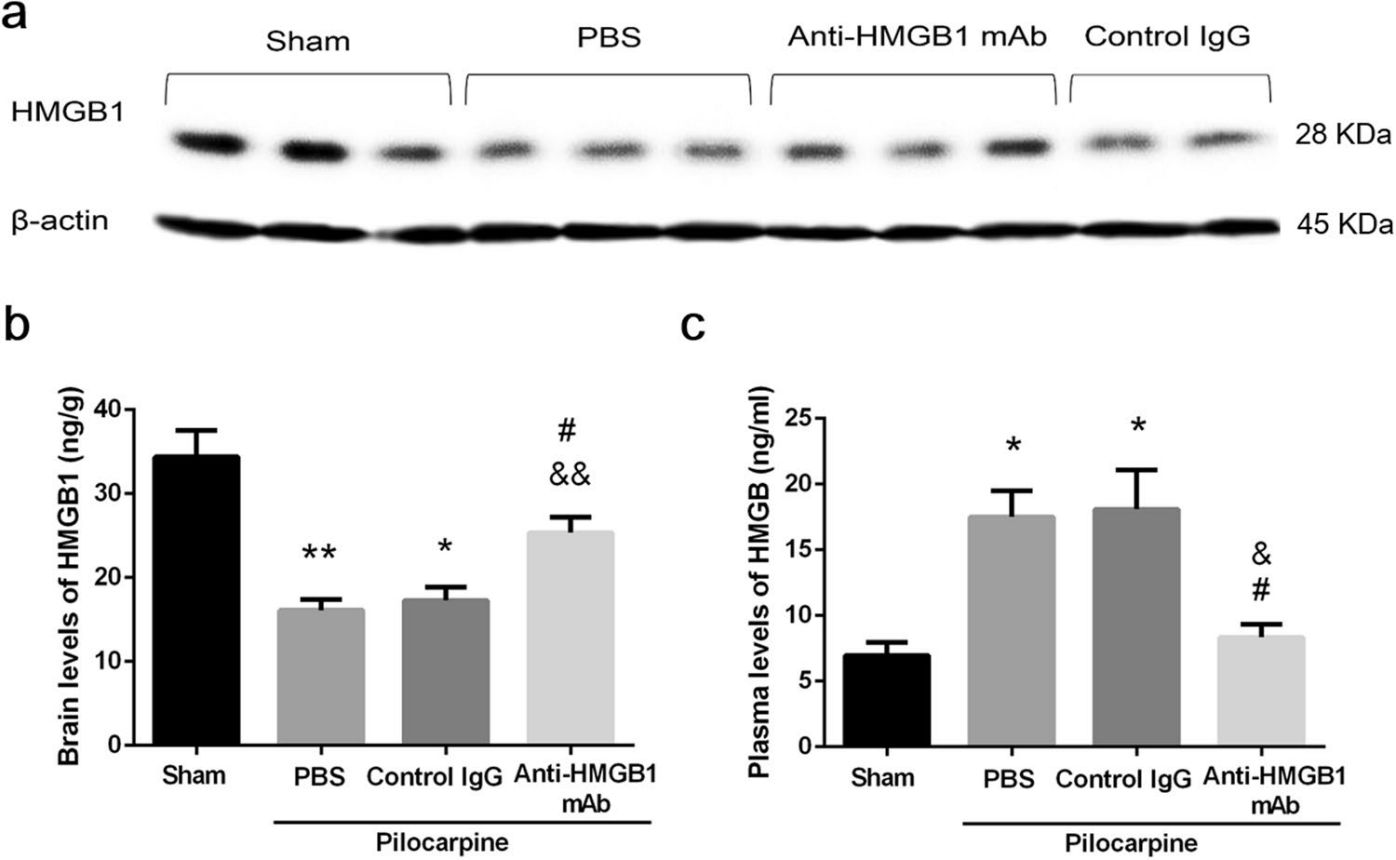
Dynamic changes of HMGB1 content in brain and plasma in pilocarpine-induced acute status epilepticus. The content of HMGB1 in the cerebrum was measured by Western blotting 4 h after anti-HMGB1, control IgG or PBS injection. The β-actin was used as a reference protein. Representative results are shown for each group of 2–3 mice (**a**). The brain levels of HMGB1 are shown as the means ± SEM of 9 mice. *p < 0.05, **P < 0.01 compared with the sham control, ^&&^p < 0.01 compared with the PBS control, ^#^p < 0.05 compared with the control IgG group (**b**). Plasma levels of HMGB1 were determined by ELISA at 4 h after onset of seizure . The results are shown as the means ± SEM of 9 mice. *p < 0.05 compared with the sham control, ^&^p < 0.05 compared with the PBS control, ^#^p < 0.05 compared with the control IgG group (**c**).

HMGB1 translocation from the brain into peripheral blood is increasingly recognized as a crucial reason for the inflammation response during epileptogenesis. In this research, the amounts of HMGB1 in the brain and plasma were detected by Western blot and ELISA. Acute status epilepticus led to a significant reduction of the amount of HMGB1 in the cerebrum due to the HMGB1 translocation (Fig. 2a). However, treatment with anti-HMGB1 mAb partially inhibited the HMGB1 translocation and increased the amount of HMGB1 in mice in an acute status epilepticus compared with the PBS and control IgG groups. At the same time, the plasma concentrations of HMGB1 were significantly higher in the PBS and control IgG groups than in the sham and anti-HMGB1 mAb-treatment groups, indicating an increase in HMGB1 translocation into the peripheral circulation (Fig. 2c). Anti-HMGB1 mAb significantly suppressed the HMGB1 levels in plasma.

These results clearly demonstrated that anti-HMGB1 mAb treatment suppressed the acute status epilepticus-induced translocation of HMGB1 into the peripheral circulation.

### Evaluation of HMGB1 translocation in the CA1 hippocampal and cerebral cortex regions by immunofluorescence staining

**Figure 3:**
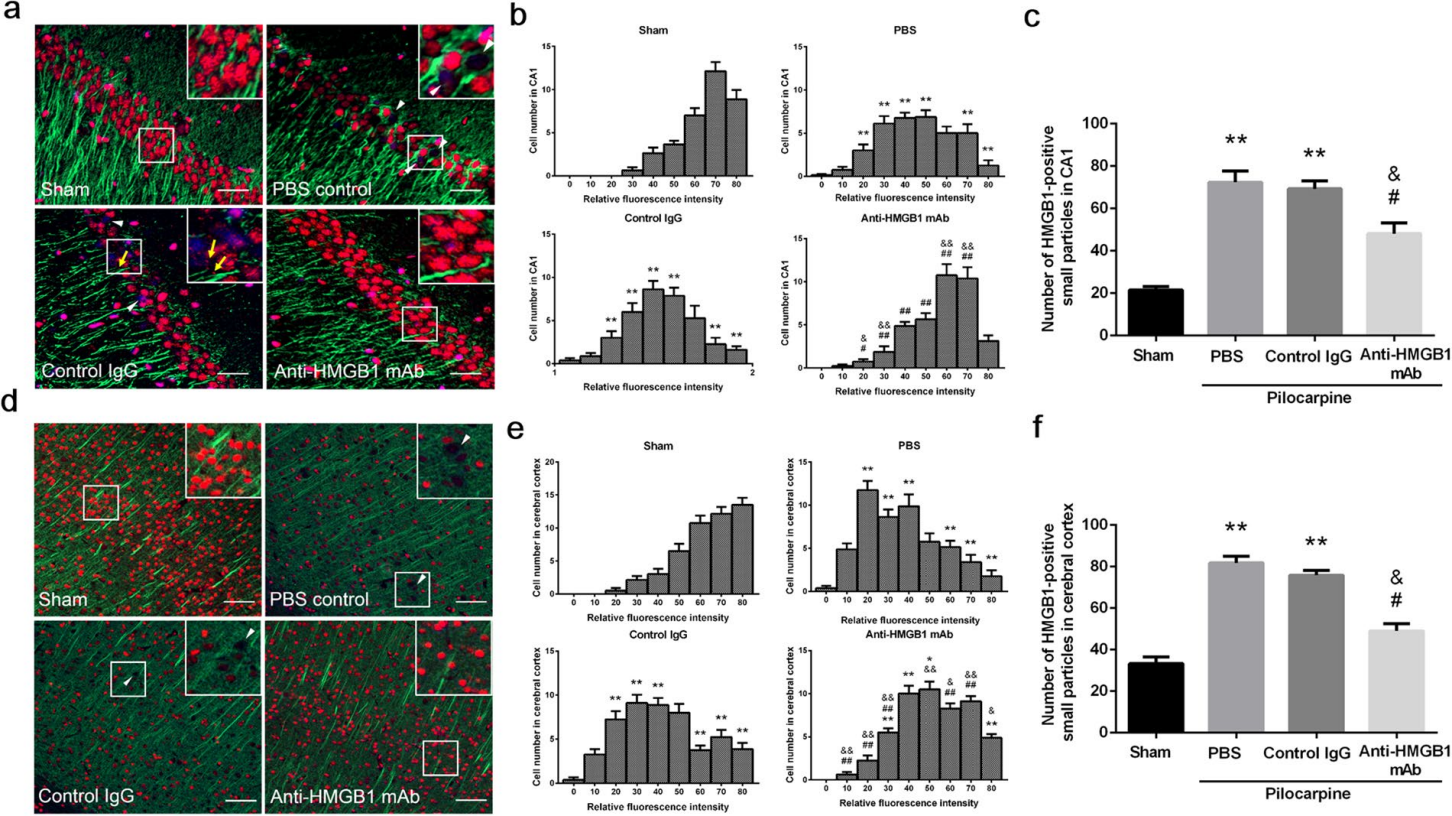
The translocation and release of HMGB1 in neurons in pilocarpine-induced acute status epilepticus. Anti-HMGB1 mAb, control IgG or PBS was administered to stage 5 epileptic mice, and the brains were fixed 4 h later. The CA1 hippocampal region (**a**) and cerebral cortex (**b**) were double-stained with anti-HMGB1 and anti-MAP2 antibodies, followed by Alexa Fluor 555-labeled and Alexa Fluor 488-labeled secondary antibodies, respectively. Scale bars equal 30 μm (**a**) and 50 μm (**d**), respectively. Arrowheads (white) indicate the neurons with marked decrease in HMGB1 fluorescence intensity in nuclei. Arrows (yellow) indicate the HMGB1-positive small particles released from nuclei (**a**). The fluorescent intensity of HMGB1 in nuclei was graded into 9 levels and the cell numbers with the indicated fluorescent levels were counted (**b**,**e**). The total number of HMGB1-positive small particles were also counted in each group (**c**,**f**). The results are shown as the means ± SEM of 8 mice. **p < 0.01 compared with the corresponding sham control. ^&^p < 0.05, ^&&^p < 0.01 compared with PBS control. ^#^p < 0.05, ^##^p < 0.01 compared with the control IgG group (**b**,**c**,**e**,**f**).

Under normal conditions in the present study, almost all CA1 neurons showed HMGB1-immunoreactivity in the nucleus (Fig. 3a). However, pilocarpine treatment caused translocation of HMGB1 from the nuclei, resulting in decrease in HMGB1 fluorescence intensity in the nuclei and increase in the number of released HMGB1 particles (yellow arrows) out of the nuclei (Fig. 3a,b,c). We graded the immunofluorescence intensity in each nuclei into 9 levels and counted the cell numbers with the indicated fluorescence levels. Figure 3b summarized the results in CA1 hippocampal region. Apparently, the cell numbers with high HMGB1 immunofluorescence in nuclei decreased in pilocarpine-injected PBS and control IgG groups compared with those in sham group whereas the cell numbers with lower HMGB1 immunofluorescence in nuclei increased in pilocarpine-injected groups. Treatment with anti-HMGB1 mAb not only inhibited the distribution pattern of histogram to the left direction by pilocarpine but also the number of small granules outside nuclei (Fig. 3b,c). Similar results were found in the cerebral cortex neurons (Fig. 3d,e,f).

These results strongly suggested that HMGB1 translocation and release occurred in the CA1 and cerebral cortex regions under an acute epileptic state, and anti-HMGB1 mAb treatment could inhibit the HMGB1 translocation into the cytoplasm and extracellular space (Fig. 3).

### Histochemical staining

**Figure 4:**
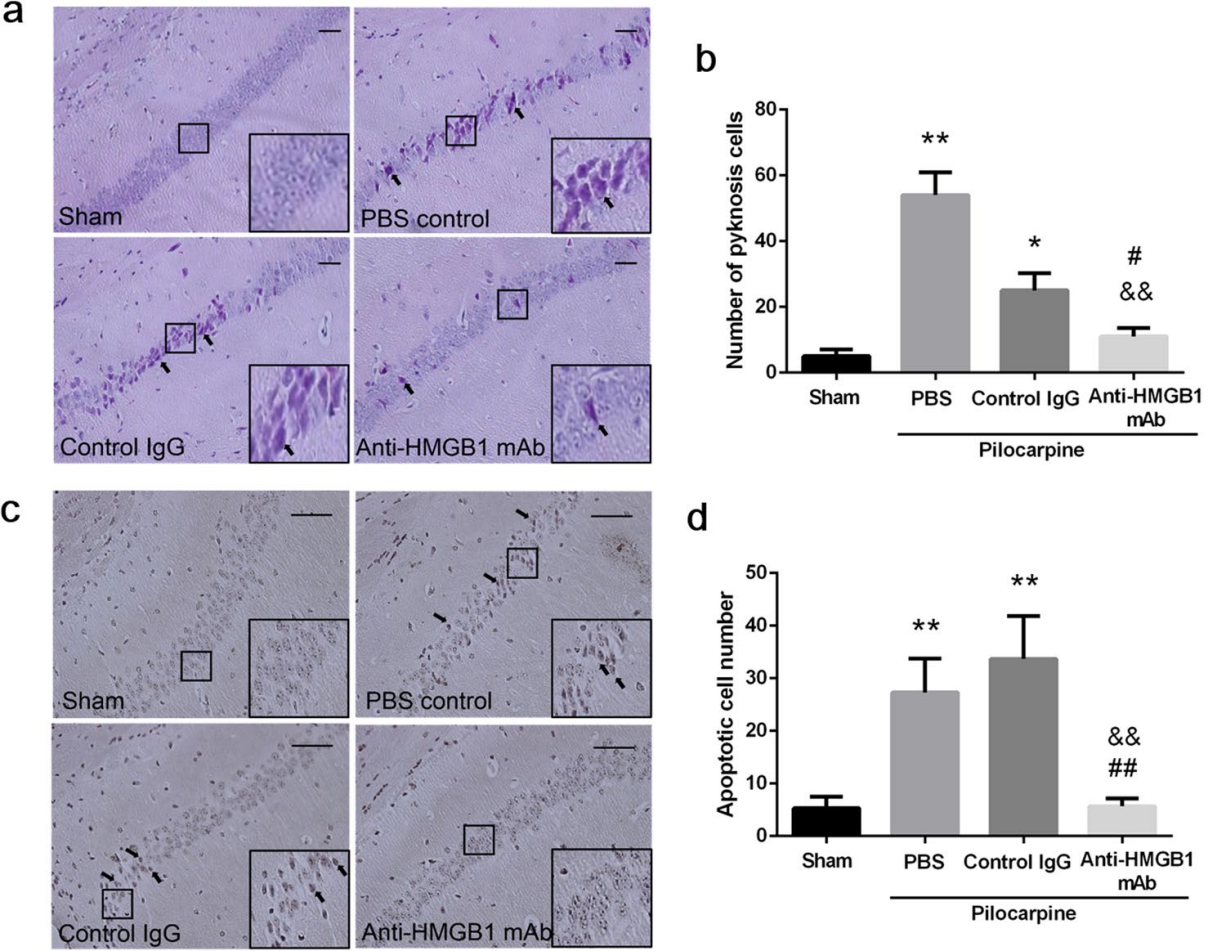
Histological analysis of neuron degeneration and apoptosis in the CA1 hippocampal region in acute status epilepticus mice. The brains were fixed with formalin at 4 h after anti-HMGB1, control IgG or PBS administration, and paraffin-embedded sections were stained with hematoxylin-eosin. Arrows indicate typical pyknotic cells. Scale bars equal 50 μm. *p < 0.05, **p < 0.01 compared with the sham. ^&&^P < 0.01 compared with the PBS control. ^#^p < 0.05 compared with the control IgG group (**a**). The number of pyknotic cells was counted in each mouse and the results are shown as the means ± SEM of 8 mice (**b**). Apoptotic cells were detected by TUNEL staining. The arrows indicate the apoptotic cells (**c**). The number of apoptotic cells in the CA1 region was counted in each mouse and the results are shown as the means ± SEM of 8 mice. **p < 0.01 compared with the sham control. ^&&^p < 0.01 compared with the PBS control. ^##^p < 0.01 compared with the control IgG group (**d**).

In our previous study, we observed degenerated neurons in the hippocampus after brain injury^22^, and cytoplasm pyknosis and cell shrinking were the two main morphological changes. In this experiment, we found a similar type of cells in the CA1 hippocampal region after acute status epilepticus, and some of these pyknotic cells showed the condensed hematoxylin staining. Acute epileptic state increased the number of pyknotic and shrunk cells, but anti-HMGB1 mAb treatment significantly reduced the number of pyknotic cells. The pyknotic cells are indicated by the black arrows in Fig. 4a. The number of pyknotic cells was significantly decreased after anti-HMGB1 mAb treatment (Fig. 4b).

Apoptotic cells are shown as a dark-brown color by TUNEL staining, and these cells could be detected in both the PBS control and control IgG groups. However, there were fewer apoptotic cells in the CA1 hippocampal region after anti-HMGB1 mAb treatment. In the sham group, no prominent apoptotic cell morphology was observed (Fig. 4c), while anti-HMGB1 mAb dramatically reduced the number of apoptotic cells after acute status epilepticus. From these results, it could be concluded that anti-HMGB1 mAb conferred protective effects on CA1 hippocampal neurons under an acute epileptic state (Fig. 4).

### Morphological changes of astrocytes and microglial cells in the CA1 and dentate gyrus hippocampal regions by immunofluorescence staining

**Figure 5:**
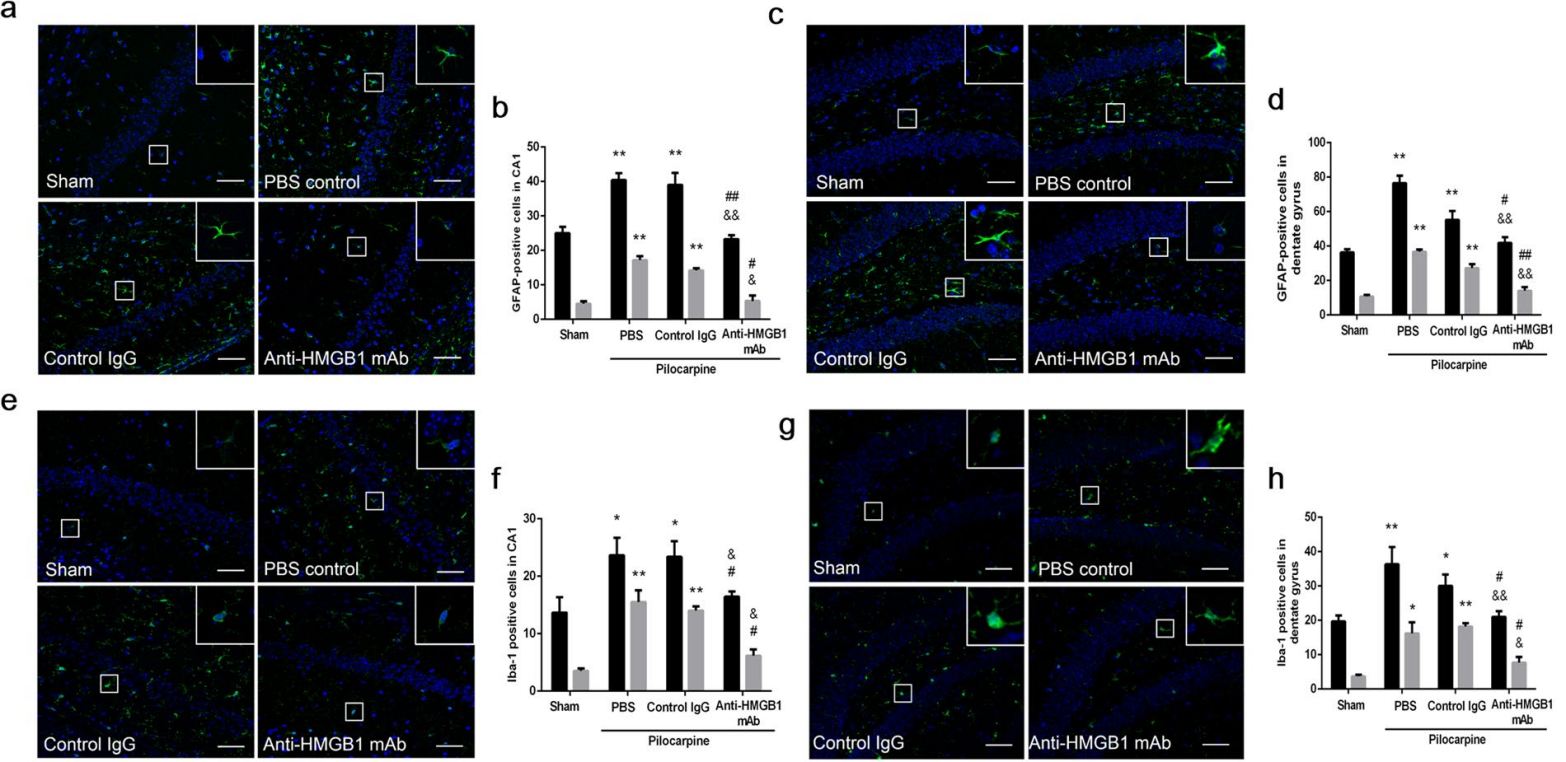
Morphological changes of astrocytes and microglia cells in the hippocampus and dentate gyrus of mice under acute status epilepticus. Anti-HMGB1 mAb, control IgG or PBS was immediately administered to mice reached Racine stage 5 and sacrificed 4 h later. The brain sections were stained with anti-GFAP (green) and DAPI (blue). The total number and activated astrocytes in the CA1 (**a**) and dentate gyrus (**c**) were counted in each group and the results are shown as the means ± SEM of 8 mice. Black bar showed the total number of astrocytes and the gray bar showed the number of activated astrocytes. **p < 0.01 compared with the sham control. ^&^p < 0.05, ^&&^p < 0.01 compared with the PBS control. ^#^p < 0.05, ^##^p < 0.01 compared with the control IgG group (**b**). **p < 0.01 compared with the sham control. ^&&^p < 0.01 compared with the PBS control. ^#^p < 0.05, ^##^p < 0.01 compared with the control IgG group (**d**). The brain sections were stained with anti-Iba1 (green) and DAPI (blue). The total number and active/amoeboid Iba1-positive cells were counted in the hippocampus (**e**) and dentate gyrus (**g**) in each group and the results are shown as the means ± SEM of 8 mice. *p < 0.05, **p < 0.01 compared with the sham control. ^&^p < 0.05 compared with the PBS control. ^#^p < 0.05 compared with the control IgG group (**f**). **p < 0.01, **p < 0.05 compared with the sham control. ^&^p < 0.05, ^&&^p < 0.01 compared with the PBS control. ^#^p < 0.05 compared with the control IgG group (**h**). Scale bars equal 50 μm.

Epilepsy is usually accompanied by the activation of astrocytes and microglial cells, and these activations promote inflammatory cytokine release and accelerate the inflammation response. In response to seizure, a proportion of reactive astrocytes and microglia cells proliferate, and this process could increase the number of astrocytes and microglia cells at the epileptic lesion site. The typical reactive phenotype of astrocytes characterized by the distinct thickening and hypertrophy could be accompanied by up-regulated GFAP expression. Activated microglia cells show fewer ramified process with the soma enlargement or “bushy” phenotypes. Hence, we counted the total number and activated astrocytes and microglial cells number under the acute epileptic state in this model. Immunohistochemical study revealed that the numbers of activated astrocytes and microglia had increased in the CA1 region of epileptic mice after treatment with PBS or in the Control IgG mice. Anti-HMGB1 mAb treatment significantly reduced the number of astrocytes and microglial cells while simultaneously maintaining or recovering their original cell shape (Fig. 5a,b,e,f). A similar trend was seen in the dentate gyrus region (Fig. 5c,d,g,h).

The neutralization of HMGB1 by anti-HMGB1 mAb significantly suppressed the acute status epilepticus-caused astrocytes and microglia cells activation.

### Evaluation of IL-1β expression in the CA3 region and thalamus under an acute status epilepticus

**Figure 6:**
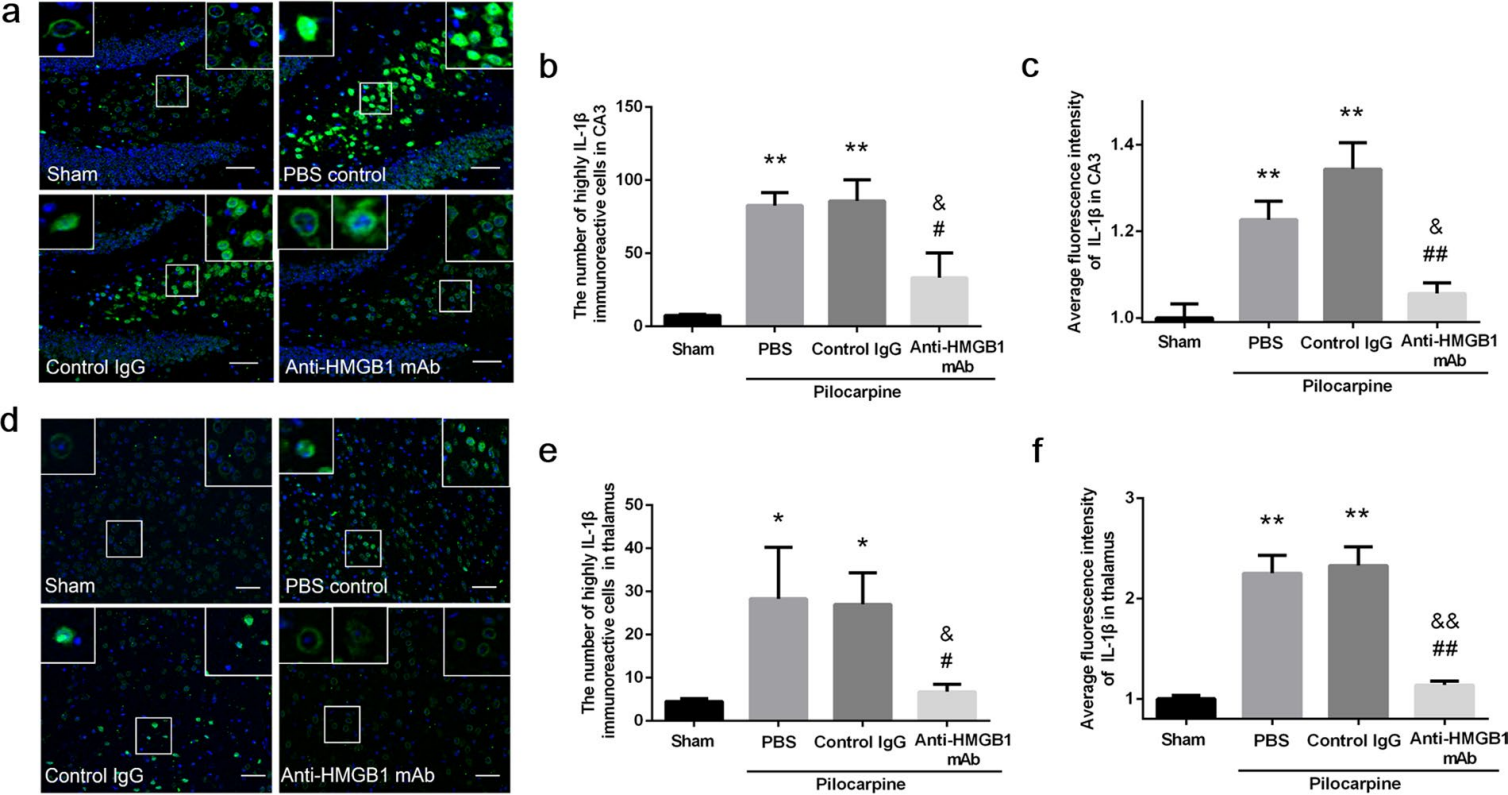
Expression of IL-1β in the CA3 hippocampal region and the thalamus of mice under acute status epilepticus. Anti-HMGB1 mAb, control IgG or PBS was administered intravenously when mice reach Racine stage 5 by pilocarpine administration and sacrificed 4 h later. The brains were fixed by formalin and paraffin-embedded sections were stained with anti-IL-1β antibody. The hippocampal CA3 (**a**) and thalamus region (**d**) were under observation. The number of highly IL-1β immunoreactive cells (**b**) and the fluorescence intensity (**c**) were analyzed in hippocampus CA3. **p < 0.01 compared with the sham group. ^&^p < 0.05 compared with the PBS group. ^##^p < 0.01, ^#^p < 0.05 compared with the control IgG group. The results are shown as the means ± SEM of 8 mice. The number of highly IL-1β immunoreactive cells (**e**) and the fluorescence intensity (**f**) were analyzed in thalamus. The results are shown as the means ± SEM of 8 mice. **p < 0.01, *p < 0.05 compared with sham group. ^&&^p < 0.01, ^&^p < 0.05 compared with the PBS group. ^##^p < 0.01, ^#^p < 0.05 compared with the control IgG group (**d**). Scale bars equal 50 μm.

Since IL-1β is thought to be a pro-inflammatory cytokine that participates in the regulation of immune and inflammatory responses. We counted the number of highly IL-1β immunoreactive cells and measured the fluorescence intensity derived from acute status epilepticus. These specific cell were shown in the left insets from the PBS control, control IgG group and in the middle inset from anti-HMGB1 mAb group (Fig. 6a,d). We found that IL-1β was expressed at very low levels in the nuclei of CA3 pyramidal cells and thalamic cells in intact mice. The expression of IL-1β in intact mice was restricted to the perinuclear regions, but IL-1β in the PBS and control IgG mice was more diffusely distributed inside the cells, while at the same time the intensity of staining in the PBS and control IgG mice was much higher than in the sham group (Fig. 6a,d).

Anti-HMGB1 treatment also remarkably inhibited the number and the fluorescence intensity of highly IL-1β- immunoreactive cells in both the CA3 and thalamus (Fig. 6b,c,e,f).

### Determination of inflammation-related molecules in the hippocampus and cerebral cortex under an acute epileptic state

**Figure 7:**
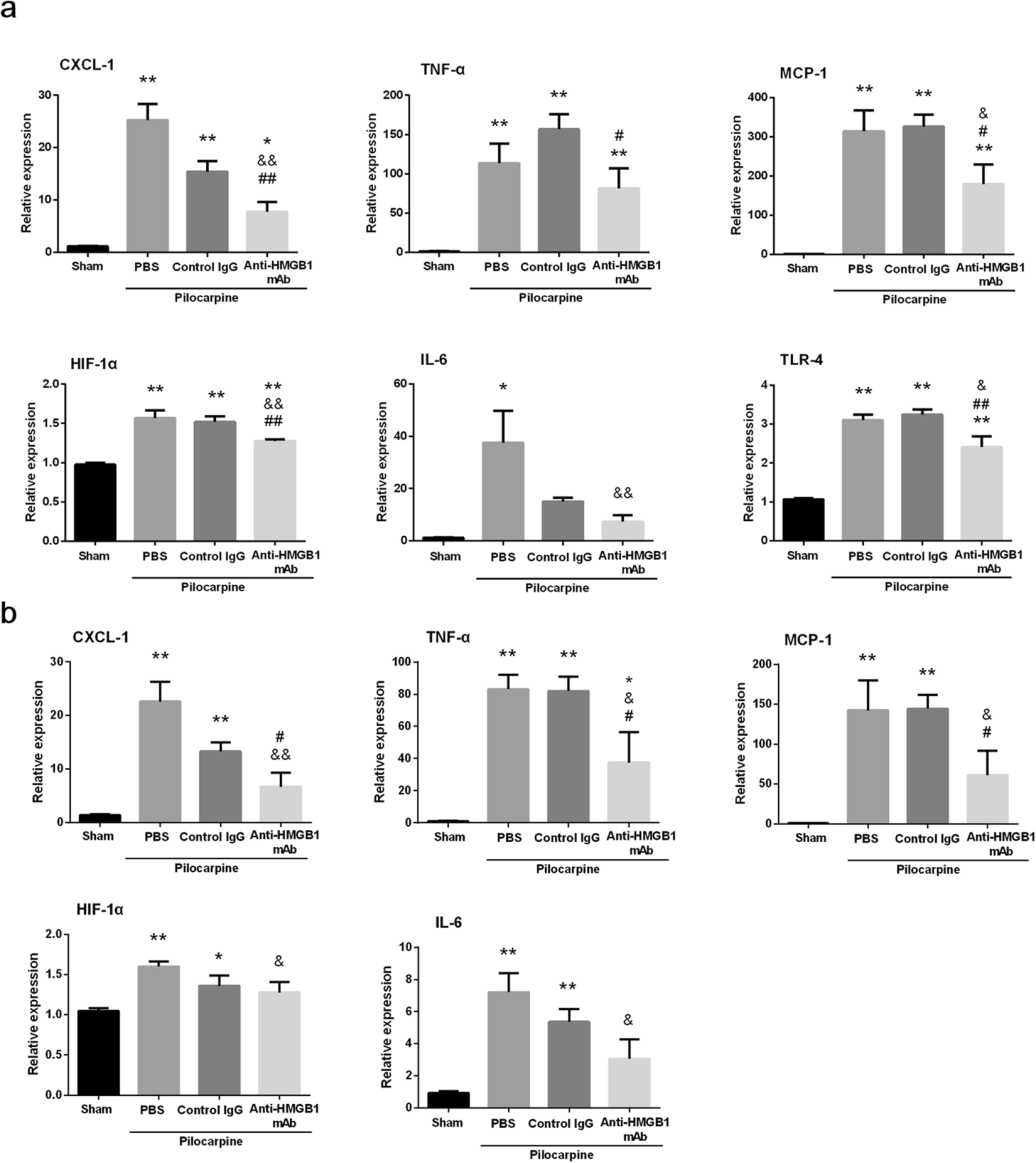
Determination of the expression of inflammation-related molecules by quantitative real-time PCR in the hippocampus (**a**) and cerebral cortex (**b**) in mice with pilocarpine-induced acute status epilepticus. Anti-HMGB1, control IgG or PBS was administered intravenously when mice reached Racine stage 5 after pilocarpine. Brain samples were collected 24 h after antibody administration. The results were normalized to the expression of GAPDH and are expressed as the means ± SEM of 11 mice. *p < 0.05, **p < 0.01 compared with the sham group. ^&^p < 0.05, ^&&^P < 0.01 compared with the PBS control. ^#^p < 0.05, ^##^p < 0.01 compared with the control IgG group (**a**,**b**).

Inflammation-related molecules were produced and released from damaged cells during the period of epilepsy, followed by the exacerbation of BBB disruption and inflammation. The expressions of hippocampal MCP-1, CXCL-1, TNF-α, HIF-1α, and TLR-4 were significantly upregulated in the PBS and Control IgG-treatment groups, whereas anti-HMGB1 antibody treatment suppressed the hippocampal expression of these inflammation-related factors (Fig. 7a). A similar trend was observed in the cerebral cortex region (Fig. 7b).

### Behavior and latency test

**Figure 8:**
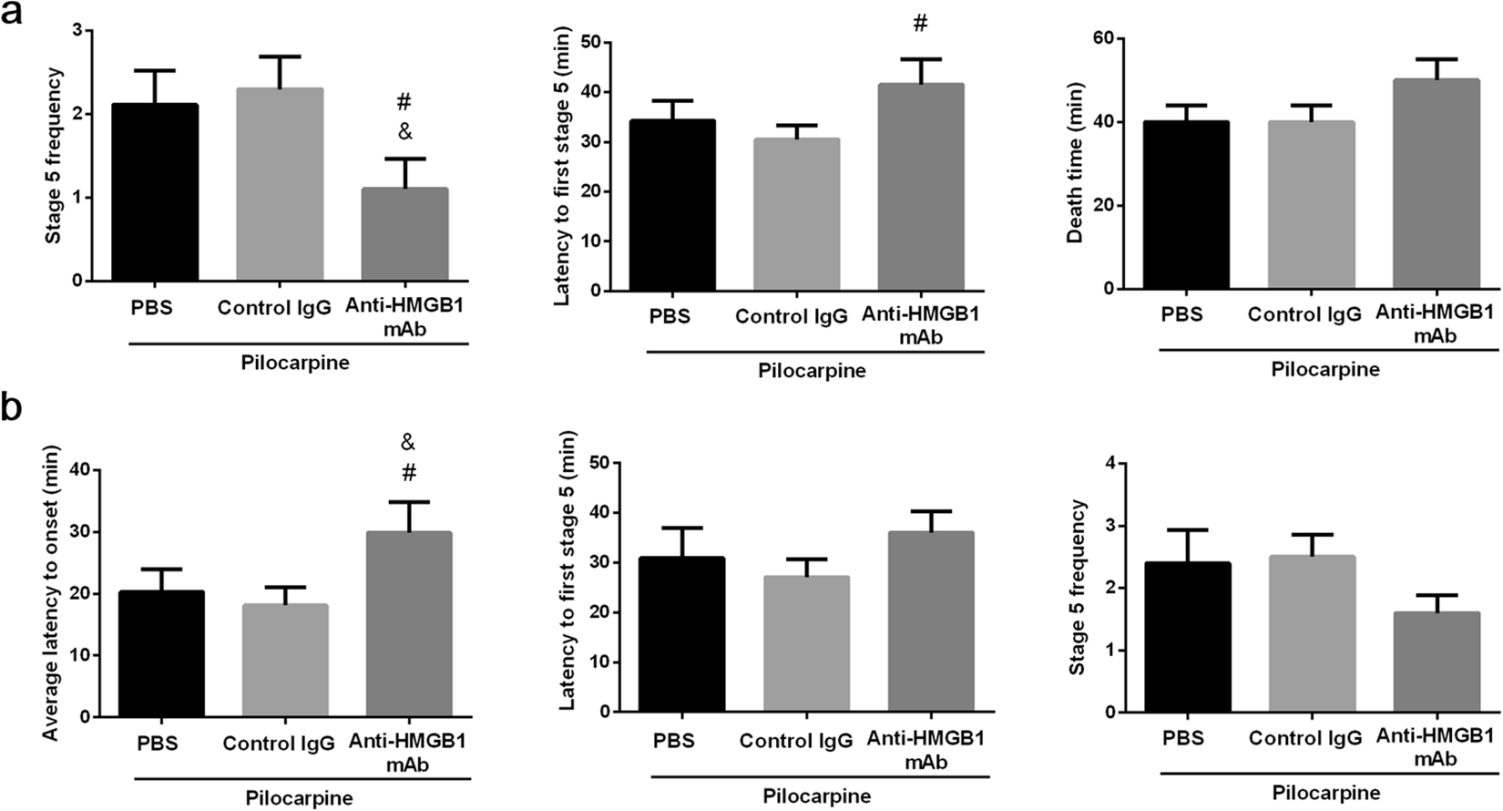
The pre-treatment and post-treatment of anti-HMGB1 mAb on acute seizure behavior. Pre-treatment: Anti-HMGB1 mAb, control IgG or PBS was administered intravenously 4 h before pilocarpine (350 mg/kg, i.v.) injection. All data were collected in 60 min after pilocarpine treatment. The frequency of seizure stage 5, latency to stage 5 and the time to death were determined on each mouse. The results are shown as the means ± SEM of 9 mice. ^&^p < 0.05, compared with the PBS control. ^#^p < 0.05, compared with the control IgG group (**a**). Post-treatment: anti-HMGB1 mAb, control IgG or PBS were immediately injected after pilocarpine injection, and behavior test were observed under 60 min observation. The latency to onset and stage 5 and the frequency of stage 5 were determined on each mouse. The results are shown as the means ± SEM of 11 mice. ^&^p < 0.05, compared with the PBS control. ^#^p < 0.05, compared with the control IgG group (**b**).

Pretreatment of anti-HMGB1 mAb 4 h before pilocarpine treatment reduced the Racine stage 5 frequencies, and increased both the latency to stage 5 and the time to death (Fig. 8a). Moreover, anti-HMGB1 mAb treatment immediately after pilocarpine injection increased the latency to onset and to the stage 5, while at the same time lowering the stage 5 frequency (Fig. 8b).

## Discussion

The cholinergic agonist pilocarpine has been widely used experimentally to mimic SE in rodents. Pilocarpine-induced epilepsy is triggered primarily by the activation of M1 muscarinic receptors in the central nervous system (CNS), and the seizures are maintained by N-Methyl-D-aspartate (NMDA) receptor activation^25^. Systemic administration of pilocarpine in rodents reproduces the principle features of human TLE^26^, i.e., limbic seizures, secondary generalized seizures and SE that lasts for several hours^26, 27^.

BBB disruption and inflammatory responses have been strongly suggested to be involved in the epileptogenic process for decades. In fact, Evans blue dye leakage after pilocarpine injection in mice was previously reported^28^ and was confirmed in the present study. Although there are many candidate molecules for induction of the increase in BBB permeability, the fact that a specific antibody against HMGB1 significantly inhibited the BBB permeability clearly demonstrated that HMGB1 is one such molecule and plays an important role in the increase in BBB permeability in the acute phase of epilepsy. Moreover, we found that the BBB disruption was exacerbated after the administration of exogenous HMGB1, implying the possible influence of systemic inflammation on vascular damage in the brain, which has also been reported in experimental stroke^29^ and brain trauma^22^ in addition to status epilepticus^30^.

Taken together, the translocation of HMGB1 in neurons, decreased HMGB1 levels in the brain and increased HMGB1 level in plasma in mice with pilocarpine-induced epilepsy support the notion that HMGB1 was translocated and released from neuronal nuclei into the surrounding areas, including the blood stream. Choy *et al*.^31^ and Patterson *et al*.^32^ also revealed that HMGB1 could be translocated from nuclei to the cytoplasm in neuronal cells at 1 and 3 h after experimental febrile status epilepticus. The mobilization pattern and dynamics of HMGB1 in mice with pilocarpine-induced epilepsy were quite similar to those observed in ischemia^18, 21^ trauma^22^ and neurotoxin^19^ induced injuries. Moreover, the inhibition of HMGB1 mobilization by anti-HMGB1 was common to different types of brain injuries, suggesting the existence of HMGB1-induced mechanisms of HMGB1 release in all brain injuries examined. Recent studies have suggested that a chemical modification of HMGB1 is responsible for the initiation of translocation^33, 34^. Therefore, there might be a common signaling mechanism leading to chemical modification among the different insults. In a previous study^21^, our group demonstrated the direct effects of HMGB1 on vascular endothelial cells and pericytes leading to contractile responses of these cells and the BBB breakdown. Based on these findings, it was speculated that both HMGB1 release into the CNS and HMGB1 release into the peripheral blood stream after pilocarpine injection contributed to the induction of BBB breakdown.

Li *et al*. reported that microglial activation in the hippocampus was suppressed after an intraventricular injection of anti-HMGB1 antibody in status epilepsy induced by intraventricular KA injection^35^. The activation of astrocytes and microglia in pilocarpine-induced acute status epilepticus was also confirmed by the increase in the number of cells and the activation-specific cell shape not only in the hippocampal but also the cerebral cortex regions. This activation of cells was significantly inhibited by the systemic administration of anti-HMGB1 mAb. Thus, the treatment of anti-HMGB1 mAb not only decreased BBB breakdown but also suppressed the activity of intrinsic inflammatory cells in the brain. Consistent with these findings, the determination of the expression of a representative pro-inflammatory cytokine, IL-1β, by immunohistochemistry clearly revealed the upregulation of IL-1β in neurons in the hippocampus and thalamus in pilocarpine-induced acute status epilepticus in mice. The up-regulation of IL-1β expression was once again significantly inhibited by the anti-HMGB1 administration. Furthermore, quantitative RT-PCR revealed that the up-regulated expressions of MCP-1, TNF-α, IL-6 and CXCL-1 were all suppressed by anti-HMGB1 mAb treatment. Since these cytokines are suggested to be involved in epileptogenesis through the amplification of inflammation^36^ and neural network remodeling in kindling^37^ as well as kainate-induced model^38^, it is likely that anti-HMGB1 treatment can inhibit the downstream processes induced by these cytokines. Among the up-regulated cytokines, IL-1β has been reported to form a complex with HMGB1, and the resultant complex increased the affinity of IL-1β to IL-1β receptors^39, 40^. Therefore, it is worth noting that the inhibitory effects of anti-HMGB1 on IL-1β expression are particularly important with regard to the suppression of enhanced and synergistic inflammation by the combination of HMGB1 and IL-1β. Moreover, the effects of HMGB1 and IL-1β appear to be very similar, as both are blocked by ifenprodil, a selective antagonist of NR2B containing NMDA receptors^41^, suggesting the involvement of glutamate downstream of HMGB1 and IL-1β action. Inhibition of inflammatory factors is becoming a novel component in the recently proposed etiologies for epilepsy^36^. However, the simultaneous suppression of several inflammatory cytokines by a single drug would be difficult. Nonetheless, anti-HMGB1 mAb would seem to be a candidate for such a drug therapy, due to its ability to inhibit the expression of a diverse range of inflammation-related molecules.

*Maroso et al*. demonstrated that microinjection of exogenous HMGB1 into the hippocampus in mice dose-dependently enhanced the seizures induced by kainate through the stimulation of TLR-4. They also suggested the involvement of RAGE, another receptor for HMGB1^42^ in kainate-induced seizures in mice^43^. Since we observed the release of HMGB1 from hippocampal pyramidal cells in the present study, and pilocarpine was reported to induce glutamate release^44^, it is quite likely that the released HMGB1 produced the synergistic effects with excitatory glutamate leading to induction of seizures^24^ as well as neuronal apoptosis^45^. The inhibitory effects of anti-HMGB1 on TLR-4 expression further support the beneficial effects of anti-HMGB1 treatment. In fact, anti-HMGB1 treatment significantly inhibited the apoptosis of pyramidal cells. Thus, it was speculated that anti-HMGB1 treatment could contribute not only to the suppression of BBB disruption and brain inflammation but also to the prevention of neuronal cell death.

Epilepsy has a complex pathophysiology. Recurrent seizures have been shown to result in the progressive development of susceptibility to sub-convulsive stimuli^46^. To induce such a state, reorganization of the neuronal network and phenotypic changes in glial cells may take place, in association with BBB breakdown, disorder of the neurovascular unit, activation of microglia and astrocytes, and up-regulation of inflammation-related molecules, including cytokines. Our findings that anti-HMGB1 suppressed the important underlying processes of epilepsy, such as BBB breakdown, inflammation and neuronal apoptosis, strongly suggested that the mobilized HMGB1 from neuronal nuclei during seizure may in turn facilitate a diverse range of events in obtaining epileptogenesis. Overall, the treatment with anti-HMGB1 mAb in the present study prolonged the latency of stage 5 and reduced the frequencies of seizure stage 5 in pilocarpine-induced acute epilepsy. These anti-epileptic effects of anti-HMGB1 mAb are probably due to the inhibition of BBB disruption, inflammatory responses and neuronal cell death. In other words, it is likely that HMGB1 mediates the recurrence and progression of epilepsy through the damage of BBB and induction of inflammation. The A box domain of HMGB1, an alternative to inhibit HMGB1 action as an antagonist for RAGE, has been administered intracerebroventricularly in many cases. The route of administration and half-life of the A box domain would limit its clinical use. Also, the efficacy of some small molecules which have been suggested to inhibit HMGB1 were limited. In contrast, intravenous injection of antibody is simple and convenient and usually has a longer half-life than HMGB1 A box domain and the small molecules antagonist^24^.

It is well-known that traumatic injury to the brain frequently causes post-traumatic epilepsy. Acute injury to the cerebral cortex by fluid percussion in rats produced a marked brain swelling that was strongly inhibited by administration of anti-HMGB1 mAb after the onset^22^. The analysis of a mechanism for protecting the BBB in this animal model clearly showed that anti-HMGB1 strongly inhibited the translocation and release of HMGB1 from neuronal nuclei and the subsequent cascade of inflammatory events very efficiently^22^. Thus, the treatment with anti-HMGB1 not only protected against acute brain damage but also suppressed the epileptogenic responses in the brain. At present, we do not have any knowledge about the spontaneous and continuous release of HMGB1 within a possible epileptic focus in the subacute or chronic phase of brain injury. Based on the present results, this type of HMGB1 release should accelerate the epileptogenesis. Further studies will be needed to examine this possibility.

In conclusion, we demonstrated for the first time that intravenous treatment with neutralizing anti-HMGB1 mAb conferred protective effects on neuronal apoptosis, in association with an inhibition of HMGB1 release, protection of the BBB and inhibition of inflammation in a very-acute-phase of status epilepticus induced by pilocarpine. These effects of anti-HMGB1 mAb as a whole appear to lead to the prolongation of seizure latency and seizure frequencies in pilocarpine-induced acute status epilepticus. Thus, anti-HMGB1 therapy may provide a novel strategy for controlling the epileptogenesis.

## Materials and Methods

### Animals

Female C57BL/6 N mice (Charles River Laboratory, Japan, Yokohama, Japan) weighing 18 to 23 g were used. Mice were housed in isolator cages with free access to food and water in a room maintained at a constant temperature of 23 °C on a 12-h light-dark cycle. The experimental protocols are described in Fig. 1. All animal experiments were approved (OKU-2014-96) by the Institutional Animal Care and Use Committee of Okayama University, and performed according to the guidelines of Okayama University on animal experiments.

### Model of pilocarpine-induced epileptic state

Mice were pre-treated with methyl-scopolamine bromide (Sigma-Aldrich Co., St. Louis, MO, USA) (1 mg/kg, i.p.) followed by pilocarpine (Wako, Japan) (350 mg/kg or 300 mg/kg, i.p.) 30 min later. Anti-HMGB1 mAb (#10–22, immunoglobulin G (IgG2a) subclass, 1 mg/kg, i.v.), class-matched control IgG mAb (anti-keyhole limpet hemocyanin, 1 mg/kg, i.v.), or PBS was administered intravenously 1 min after first stage 5 seizure. The average time interval between pilocarpine injection and first stage 5 was 24 ± 1.9 min. The Racine stage^47^ was used as a criterion. Mice under Racine stage 5 seizure were excluded from this experiment. Mice were assigned randomly to each group. Sound and light stimulations were prohibited during the experiment. The sham group received a comparable intravenous injection of 10 mM PBS. A heating pad was used to maintain the body temperature of mice at 37 ± 0.5 °C.

**Figure 9:**
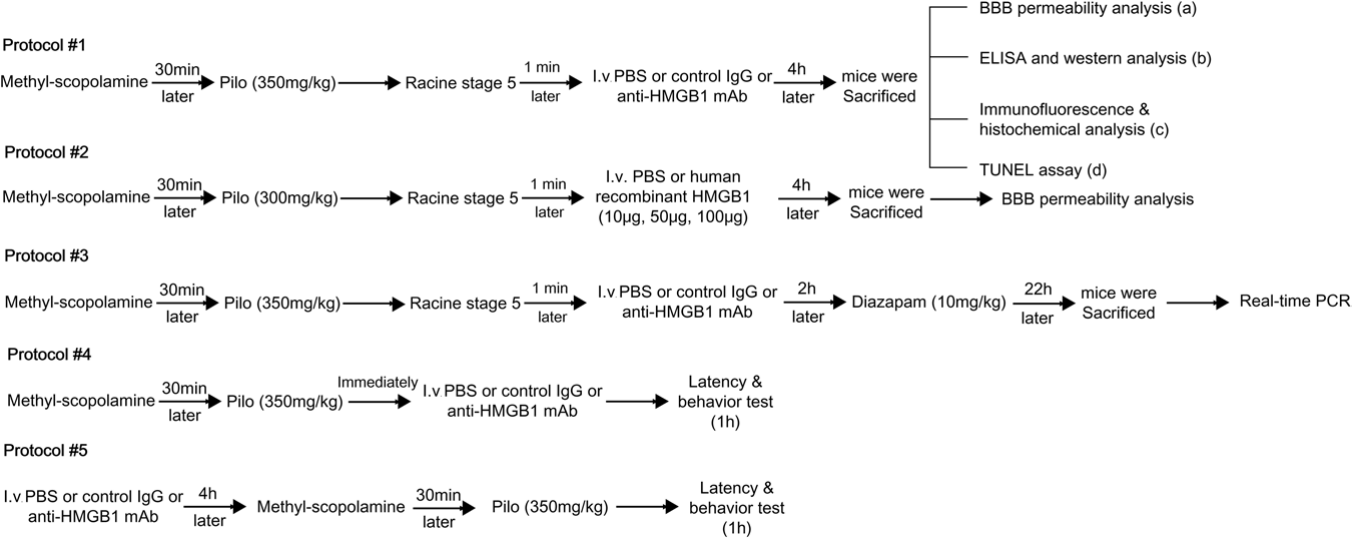
A schematic of the experimental design, drug doses and subsequent processes. Protocol #1: Racine stage 5 seizures were induced by pilocarpine which were then treated with PBS, control IgG or anti-HMGB1 mAb for 4 h, then sacrificed, and examined for the BBB permeability, the translocation of HMGB1 (**a**,**b**,**c**), and the apoptosis cells under acute status epilepticus (**d**). Protocol #2: Mice were administered 10, 50, or 100 μg of human recombinant HMGB1 for 4 h under status epilepticus condition, then BBB permeability was determinated by Evans blue again. Protocol #3: Epileptic mice were treated with PBS or control IgG or anti-HMGB1 mAb for 24 h, then subjected to RT-PCR analyses. Protocol #4: Mice were injected with pilocarpine and either PBS, control IgG or anti-HMGB1 mAb, then immediately subjected to a seizure latency test, followed by a behavior test under 1 h post-treatment. Protocol #5: Mice were pretreated with PBS, control IgG or anti-HMGB1 mAb for 4 h before pilocarpine induction followed by behavior and latency test in 1 hour.

A schematic was used for stating the experimental design, drug doses and subsequent process (Fig. 9).

### Measurement of BBB permeability

BBB permeability *(*Fig. 9, *protocol #1a)* was assessed by measuring the extravasation of Evans blue into the brain. Mice were divided into four groups: sham, PBS control group, control IgG group, and anti-HMGB1 mAb group (n = 5 in each group). Pilocarpine was administered to trigger the acute epileptic state, and then the mice that reached Racine stage 5 were selected and injected with PBS, control IgG or anti-HMGB1 mAb. Two hours later, Evans blue (100 mg/kg) was injected via the tail vein under an awake state^28^, then allowed to circulate for 2 h prior to sacrifice. The measurement of BBB permeability was performed by the Evans blue absorbance at 620 nm. See details in the Supplemental Methods.

To determine whether increases in plasma HMGB1 accelerate the BBB breakdown in pilocarpine-induced epilepsy, the recombinant human HMGB1 was immediately given via the tail vein after pilocarpine injection (Fig. 9, *protocol #2*). Mice were divided into six groups: sham group, sham with 100 μg rhHMGB1 group, pilocarpine (300 mg/kg, i.v.) plus PBS group, and pilocarpine plus 10, 50, or 100 μg rhHMGB1-treatment groups (n = 7 in each group). Mice in the sham and sham with 100 μg rhHMGB1 group received PBS instead of pilocarpine. The measurement of BBB permeability was performed as described above^18, 22, 28^.

### Measurement of HMGB1 concentrations in the cerebrum and plasma during the acute phase of status epilepticus

For determination of the HMGB1 concentration in plasma (Fig. 9, *protocol #1b*), samples were collected through the inferior vena cava under deep anesthesia with sodium pentobarbital (50 mg/kg, i.p.). After being centrifuged at 1500 g for 10 min, the sample supernatants were supplemented with protease inhibitor (Sigma) (50 μl/g) and stored at −20 °C before use. Mice were divided into four groups (n = 9 in each group). Plasma levels of HMGB1 were determined by enzyme-linked immunosorbent assay (Shino-Test Co., Sagamihara, Japan) as described previously^21^.

The HMGB1 content in the cerebrum (Fig. 9, *protocol #1b*) was determined by Western blot according to the methods described previously^21^. Brain samples were collected 4 h after injection of anti-HMGB1 mAb, control IgG or PBS under an acute status epilepticus (n = 9 mice in each group). See details in the Supplemental Methods.

### Histochemical staining and TUNEL assay

Samples were collected 4 h after anti-HMGB1, control IgG or PBS injection under acute seizure condition. The brains of the epileptic mice (Fig. 9, *protocol #1c*, *d*) were perfused with 0.9% Nacl from the left ventricle, then fixed with 10% formalin and post-fixed overnight in 10% formalin at 4 °C. The samples were then embedded in paraffin and cut at a thickness of 5 µm. Brain sections were stained with hematoxylin–eosin^18^. Changes in the morphology of cells in the CA1 hippocampal region were observed under a BZ-X700 All-In-One fluorescence microscope (Keyence, Oosaka, Japan).

Apoptotic cells were measured by the *in situ* terminal deoxynucleotidyl transferase-mediated dUTP-biotin nick end-labeling (TUNEL) method (Fig. 9, *protocol #1*) using an *in situ* Apoptosis Detection Kit (Takara, Shiga, Japan) as previously described^48^. Morphological changes of nuclei were observed under a BZ-X700 All-In-On fluorescence microscope (Keyence, Oosaka, Japan).

### Immunofluorescence staining

For immunofluorescence staining (Fig. 9, *protocol #1c*), the mice were divided into 4 groups with 8 mice in each group. Mice were treated as described in protocol #1 histochemical staining section above. Paraffin-embedded brain sections (5 μm) were stained using a mouse anti-HMGB1 mAb (R&D Systems Inc.), rabbit anti-MAP2 pAb (Santa Cruz Biotechnology Inc.) for HMGB1 detection. For astrocytes and microglia cells staining rabbit anti-GFAP pAb (Abcam, Cambridge, UK), rabbit anti-Iba-1 Ab (Wako, Osaka, Japan) were used. Rabbit polyclonal anti-IL-1β antibody (Abcam, Cambridge, UK) were used for IL-1β detection as the primary antibody. See details in the Supplemental Methods.

### Cell morphology and cell counting quantification

The H&E staining was used for identifying the pyknotic cells. The apoptotic cells were defined as those with nuclear staining with a dark-brown color using TUNEL method as described above. Under normal condition, neurons showed HMGB1- immunoreactivities in the nuclei. Reactive astrocytes usually shown as cell swelling with fine spongiform process with a greater number of main process extending from the soma and the GFAP immunoreactivity exhibited a distinct increase^49^. We set two criteria to identify activated astrocytes: 1) the thickness of main process were over 2.5 μm; 2) the length of processes from soma was more than 15 μm. Astrocytes met both criteria were identified as activated astrocyte and counted. Under resting conditions, microglia in the CNS were ramified. The resting microglial cells were characterized by a small cell soma and several branching thin processes that extend in all directions. However, when brain insult causes a transformation from ramified into activated microglia even an amoeboid form, these processes retracted microglia cell processes and become fewer and much thicker. At the same time, the size of the cell bodies will increase. Then, the criteria for activated microglia were 1) the thickness of process was over 3 μm; 2) the cell soma was oval shape and the longest diameter was longer than 12 μm; 3) the visible processes were shorter than 5 μm from the soma. Microglia cells met these three criteria were identified as activated microglia and counted. The highly IL-1β-immunoreactive cells were identified as those having more than four times higher intensity of fluorescence companied with sham average control cells in the same area.

The number of pyknotic and apoptotic cells were counted on 3 serial sections per animal and four randomly selected squares (50 × 50 μm^2^) in each section and counted the total number from these squares in the CA1 hippocampal region. The same procedure was performed when determining the HMGB1 fluorescence intensity in nuclei on the brain sections from each mice and counting the number of HMGB1-positive small particles in CA1 (30 × 30 μm^2^) and in cerebral cortex (50 × 50 μm^2^). The number of specific cells in these squares were counted by blind investigators who were unaware of the groups and statistically analyzed. The GFAP- and Iba-1-stained sections were used for counting the total number and the activated number of astrocytes and microglia cells, respectively in the square area of 100 × 100 μm^2^. The number of IL-1β specific cells were counted on 50 × 50 μm^2^ squares in CA1 and thalamus by blind investigators. The fluorescence intensity of HMGB1 and IL-1β were measured by using Image J. All the procedures abided by the preclinical research standard^50^.

### Real-time polymerase chain reaction

For analyzing the expression of inflammation-related molecules, samples were taken from the hippocampus and cerebral cortex 24 h after the treatment with anti-HMGB1 mAb, control IgG mAb or PBS under an epileptic state (n = 11 mice in each group) (Fig. 9, *protocol #3*). Total RNA was extracted from the mouse hippocampus and cerebral cortex by using an RNeasy mini kit (QIAGEN) according to the manufacturer’s protocol. The RNA was then reverse-transcribed to cDNA by a reverse transcriptase kit-RNA PCRT M Kit (AMV) Ver 3.0 (Takara, Shiga, Japan). The real-time polymerase chain reaction (RT-PCR) analysis was performed as described previously^18^ using SYBR Premix EX Taq (Takara) in a Light Cycler instrument (Roche, Indianapolis, IN) according to the manufacturer’s instructions. The sense and antisense primer sequences were described in Supplementary Table S1.

The expression of GAPDH was used to normalize cDNA levels. PCR products were analyzed using a melting curve to ascertain the specificity of amplification.

### Behavior and latency test

This experiment, we divided this part into two sections, firstly, mice were treated with PBS, control IgG (1 mg/kg) or anti-HMGB1 mAb (1 mg/kg) immediately after pilocarpine injection (n = 11 mice in each group) (Fig. 9, *protocols #4*). The time to the onset, first latency to stage 5 and the frequency of stage 5 were all recorded in an observation period of 60 min (Fig. 9, *protocol #4*).

Secondly, mice were pretreated with anti-HMGB1 mAb (1 mg/kg), control IgG (1 mg/kg) or PBS 4 h before pilocarpine (350 mg/kg) (n = 9 mice in each group) injection (Fig. 9, protocols #5). After the injection of pilocarpine, the time to the onset, first latency to stage 5, and the time to death were all recorded in an observation period of 60 min (Fig. 9, *protocol #5*). All the data were counted by an examiner who was blinded to the experimental protocol. Mice under Racine stage 5 were excluded from this experiment.

### Statistical Analysis

Statistical significances were performed using one-way analysis of variance (ANOVA) followed by *LSD* (a = 0.05) or *Dunnett* test by IBM SPSS Statistics 19.0. Results were expressed as mean ± SEM. A probability value of <0.05 was considered to be significant.

## Electronic supplementary material

Supplementary Methods and Tables
