# Supplementary material for: Therapeutic effects of anti-HMGB1 monoclonal antibody on pilocarpine-induced status epilepticus in mice
Source: Sci Rep. 2017 Apr 26;7:1179. doi: 10.1038/s41598-017-01325-y (PMC5430706; doi:10.1038/s41598-017-01325-y)
Supplement: Supplementary file 1 — Supplementary Methods and Tables [file 41598_2017_1325_MOESM1_ESM.pdf]

ONLINE SUPPLEMENT

Supplementary Methods and Tables

Therapeutic effects of anti-HMGB1 monoclonal antibody on pilocarpine-induced status epilepticus in mice

Li Fu[1]; Keyue Liu[1], MD, phD; Hidenori Wake[1], phD; Kiyoshi Teshigawara[1], phD; Tadashi Yoshino[2], MD, phD; Hideo Takahashi[3], phD; Shuji Mori[4], phD; Masahiro Nishibori[1*], MD, phD

[1]Department of Pharmacology, Okayama University Graduate School of Medicine, Dentistry, and Pharmaceutical Sciences

[2]Department of Pathology, Okayama University Graduate School of Medicine, Dentistry, and Pharmaceutical Sciences

[3]Department of Pharmacology, Kinki University School of Medicine

[4]School of Pharmacology, Shujitsu University

***Correspondence to**: Masahiro Nishibori, MD, phD.

Department of Pharmacology, Okayama University Graduate School of Medicine, Dentistry, and Pharmaceutical Sciences, Okayama 700-8558, Japan

Phone/Fax: +81-86-235-7140

Email: mbori@md.okayama-u.ac.jp

Supplementary

Methods

Measurement of BBB permeability

To determine the BBB permeability under acute status epilepticus condition, mice were under deep anesthesia with sodium pentobarbital (50 mg/kg) and were perfused via the left ventricle with 30 ml of cold saline (0.9% NaCl). Brains were immediately removed and weighed, and the cerebrum was dissected. The whole cerebrum was placed in 1 ml of 1 M potassium hydroxide, then kept overnight at 4°C and homogenized. A 0.5 ml aliquot of the homogenized suspension was mixed with 1 ml of a mixture of 0.6 M phosphoric acid and acetone (5:13), and the resulting solution was centrifuged at 17400 g for 30 min. Finally, the supernatant solution was transferred to a cuvette, and the absorbance at 620 nm was measured. Data were expressed as Evans blue ng/g wet cerebrum weight.

Measurement of HMGB1 concentrations in the cerebrum and plasma during the acute status epilepticus

Mice were anesthetized deeply with an i.p. injection of sodium pentobarbital (50mg/kg) and perfused by cold saline. Cerebra in each group were homogenized in cold radioimmunoprecipitation assay buffer (RIPA) (150 mM NaCl, 0.5% sodium deoxycholate, 0.1% SDS, 0.1% Triton X-100, and 50 mM Tris-HCl (pH 8.0)) with cocktail protease inhibitors (Sigma-Aldrich Co.). The brain homogenates were then centrifuged at 10,000 g for 20 min. The protein concentration in the supernatant was detected by Bio-Rad Protein Assay (Bio-Rad Laboratories Inc., Hercules, CA, USA). β -actin was used as a reference protein. Mouse anti-HMGB1 mAb (R&D Systems Inc., Minneapolis, MN, USA) and mouse anti-β-actin mAb (Santa Cruz Biotechnology Inc., Santa Cruz, CA, USA) were used as primary antibodies followed by HRP-conjugated goat anti-mouse IgG (MBL International Inc., Woburn, MA, USA). After washing in T-TBS, an ECL system (Thermo Fisher Scientific Inc., Rockford, IL, USA) was used to visualize the bands of HMGB1 and β -actin.

Immunofluorescence staining

Mice were anesthetized deeply with an i.p. injection of sodium pentobarbital (50mg/kg) and perfused by cold saline followed with 10% formalin. Brains were post-fixed overnight under 4ºC condition in 10% formalin and paraffin-embedded. Brain sections were cut at a thickness of 5 µm. Antigen retrieval was performed by incubating the section with 10 mM sodium citrate buffer (pH 6.0) for 10 min at 120°C. Incubation with 10% normal goat serum (Sigma-Aldrich Co.) and 1% bovine serum albumin (BSA) in 10 mM TBS for 2 h at room temperature was used to block nonspecific binding. Brain sections were stained with mouse anti-HMGB1 mAb (R&D Systems Inc.), rabbit anti-MAP2 pAb (Santa Cruz Biotechnology Inc.), rabbit anti-GFAP pAb (Abcam, Cambridge, UK), rabbit anti-Iba-1 Ab (Wako, Osaka, Japan) or rabbit polyclonal anti-IL-1β antibody (Abcam) as the primary antibody overnight. Alexa 555-labeled anti-mouse IgG (Invitrogen Co., Branford, CT, USA) and Alexa 488-labeled anti-rabbit IgG (Invitrogen Co.) were used as the secondary antibodies and incubated for 1 h at room temperature. VectorShield Hard Set Mounting Medium with DAPI (Vector Laboratories Inc., Burlingame, CA, USA) was used for mounting. Stained sections were observed under an LSM 780 confocal imaging system (Carl Zeiss Inc., Jena, Germany). The number of specific cells was counted in four randomly chosen squares.

Supplementary Table S1

Gene name	Forward sequence<br>Reverse sequence
Monocyte chemotactic protein-1 (MCP-1)	5'- GTCCCTGTCATGCTTCTGG -3'
	5'- AGGTGAGTGGGGCGTTAA -3'
interleukin- 6 (IL-6)	5'- GACCTGTCTATACCACTTCACA -3'
	5'- CTCTGGAAGTTTCAGATTGTT -3'
The chemokine (C-X-C motif) ligand 1 (CXCL-1)	5'- TGCACCCAAACCGAAGTCAT -3'
	5'- TTGTCAGAAGCCAGCGTTCAC -3'
Hypoxia Inducible Factor-1α (HIF-1α)	5'- ACCTTCATCGGAAACTCCAAAG -3'
	5'- CTGTTAGGCTGGGAAAAGTTAGG -3'
Tumor necrosis factor-α (TNF-α)	5'-GACCCTCACACTCAGATCATCCTTCT-3'
	5'-GCGCTGGCTCAGCCACTC-3'
Toll-like receptor 4 (TLR-4)	5'- TTCAGAACTTCAGTGGCTGG -3'
	5'- TGTTAGTCCAGAGAAACTTCCTG -3
Glyceraldehyde-3-phosphate dehydrogenase (GAPDH)	5'- TGACGTGCCGCCTGGAGAAA -3'
	5'-AGTGTAGCCCAAGATGCCCTTCAG -3'
